# Ultrahigh Dielectric Permittivity of a Micron-Sized Hf_0.5_Zr_0.5_O_2_ Thin-Film Capacitor After Missing of a Mixed Tetragonal Phase

**DOI:** 10.1007/s40820-025-01841-x

**Published:** 2025-07-18

**Authors:** Wen Di Zhang, Bing Li, Wei Wei Wang, Xing Ya Wang, Yan Cheng, An Quan Jiang

**Affiliations:** 1 College of Integrated Circuits and Micro/Nano Electronics Innovation, Fudan University, Shanghai, 200433 People’s Republic of China; 2 Center for Transformative Science, ShanghaiTech University, Shanghai, 201210 People’s Republic of China; 3 Shanghai Advanced Research Institute, Chinese Academy of Sciences, Shanghai, 201204 People’s Republic of China; 4 Key Laboratory of Polar Materials and Devices (MOE), Department of Electronics, East China Normal University, Shanghai, 200241 People’s Republic of China

**Keywords:** Hf_0.5_Zr_0.5_O_2_ thin film, Ultrahigh dielectric permittivity, Near-edge plasma treatment, Oxygen vacancy, Charge storage

## Abstract

Innovative use of HfO_2_-based high-dielectric-permittivity materials could enable their integration into few-nanometre-scale devices for storing substantial quantities of electrical charges, which have received widespread applications in high-storage-density dynamic random access memory and energy-efficient complementary metal–oxide–semiconductor devices. During bipolar high electric-field cycling in numbers close to dielectric breakdown, the dielectric permittivity suddenly increases by 30 times after oxygen-vacancy ordering and ferroelectric-to-nonferroelectric phase transition of near-edge plasma-treated Hf_0.5_Zr_0.5_O_2_ thin-film capacitors. Here we report a much higher dielectric permittivity of 1466 during downscaling of the capacitor into the diameter of 3.85 μm when the ferroelectricity suddenly disappears without high-field cycling. The stored charge density is as high as 183 μC cm^−2^ at an operating voltage/time of 1.2 V/50 ns at cycle numbers of more than 10^12^ without inducing dielectric breakdown. The study of synchrotron X-ray micro-diffraction patterns show missing of a mixed tetragonal phase. The image of electron energy loss spectroscopy shows the preferred oxygen-vacancy accumulation at the regions near top/bottom electrodes as well as grain boundaries. The ultrahigh dielectric-permittivity material enables high-density integration of extremely scaled logic and memory devices in the future.

**Supplementary Information:**

The online version contains supplementary material available at 10.1007/s40820-025-01841-x.

## Highlights

- Ferroelectric-to-nonferroelectric transition occurs in a micron-sized Hf_0.5_Zr_0.5_O_2_ thin-film capacitor with the generation of a giant dielectric permittivity.
- Synchrotron X-ray micro-diffraction patterns show missing of a mixed tetragonal phase in the capacitor.
- The stored charge density of the capacitor is as high as 183 μC cm^-2^ at an operating voltage/time of 1.2 V/50 ns at cycle numbers of more than 10^12^ without inducing dielectric breakdown.

**Supplementary Information:**

The online version contains supplementary material available at 10.1007/s40820-025-01841-x.

## Graphical abstract

**Figure:**
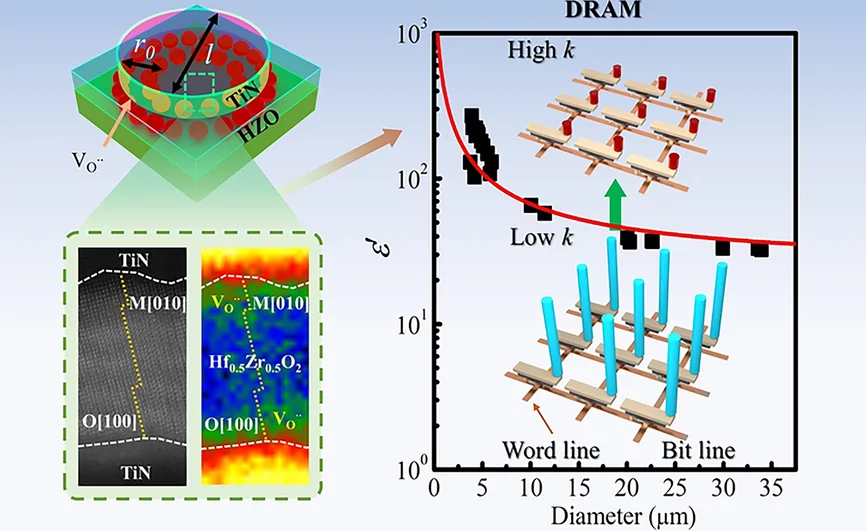

## Introduction

Compact electronics and electric power systems including high storage-density dynamic random access memory (DRAM) and energy-efficient complementary metal-oxide semiconductor (CMOS) require electrostatic capacitors to store substantial quantities of electrical charge using high dielectric-permittivity (*ε*′) materials [1–3]. The discovery of the ferroelectricity of HfO_2_-based materials offered the solution for the integration and scalability of ferroelectric based devices, and the ferroelectric capacitors could improve the energy efficiency of conventional electronics beyond fundamental limits because they overcome many of the thickness-scaling and silicon-compatibility issues [4]. Nowadays HfO_2_-based thin films can be fabricated through low-temperature atomic-layer deposition (ALD) in dielectric permittivities of 16–70 among which *ε*′ is highest for a tetragonal phase [5–14]. Both HfO_2_ and ZrO_2_ thin films are already used in everyday electronics [15], and high-*ε*′ HfO_2_/ZrO_2_ stacked layers have been proposed as a gate dielectric for sub-20 nm FinFET technology to improve the device performance significantly [16]. But the ferroelectricity of HfO_2_-based thin films is generated only from the metastable orthorhombic (O, Pca2_1_) phase between monoclinic (M, P2_1_/c) and tetragonal (T, P4_2_/nmc) phases, and their polarizations vary with the redistribution of oxygen vacancies, bonding length distortion, and the effect of mechanical stresses [17–21]. Theoretical polar features of HfO_2_ are believed to be associated with a nearly flat phonon band that causes extreme localization of electric dipoles separated by the nonpolar spacer layers in equal half-unit-cell-widths [17].

In experiments, there are many methods to improve dielectric and ferroelectric properties of HfO_2_-based thin films [16, 22, 23]: 1) hydrogen-based plasma treatment as well as point defect engineering through light-ion bombardment can increase the O phase to reduce the wakeup fatigue effect by enhancing ferroelectric polarization in comparison to Ar plasma treatment that modulates ferroelectric and antiferroelectric phases [22–25]; 2) the polar-to-nonpolar phase transitions of HfO₂-ZrO₂ thin films change their dielectric permittivity nonlinearly from 35 to 140 [26−28]; and 3) near-edge ion implantation in HfO₂-ZrO₂ thin-film capacitors can promote ferroelectric-to-nonferroelectric transition in generation of an ultrahigh dielectric permittivity of 921 [29]. In the latter, it accompanied the oxygen vacancy accumulation toward top/bottom electrodes during bipolar high-electric-field cycling. Consequent high-resolution scanning transmission electron microscopy (STEM) images showed ordered oxygen vacancies to occur within the distorted orthorhombic grains. The ultrahigh dielectric permittivity enables the realization of high charge- and energy-storage devices and energy-efficient transistors [2, 3, 8, 14, 30]. However, the cycling numbers to promote the phase transition are close to dielectric breakdown. It is wondered if the ultrahigh *ε*′ transition could occur in the virgin capacitor without high-electric-field cycling (fatigue).

Here we report the ultrahigh-*ε*′ transition to occur in an oxygen − vacancy-implanted Hf_0.5_Zr_0.5_O_2_ (HZO) ferroelectric capacitor without fatigue. The film is a mixture of O, T, and M phases, and *ε*′ increases nonlinearly upon downscaling of the capacitor size from 30 to 4.07 μm. With the further shrinkage of the capacitor size down to 3.85 μm, the ferroelectric-to-nonferroelectric transition occurs with the appearance of an ultrahigh *ε*′ of 1466. The subsequent study of synchrotron X-ray micro-diffractions shows missing of a mixed tetragonal phase after the phase transition. This discovery represents a significant advancement of the fabricated ultrahigh-*ε*′ material for applications in high-density memory, logic and energy storage devices.

## Experimental Section

### HZO Fabrication

Continuous Hf_0.5_Zr_0.5_O_2_ thin films were grown by ALD (TFS 200, Beneq) at 200 °C on a Si substrate coated with 10-nm-thick TiN bottom electrodes, where hafnium tetrachloride and zirconium tetrachloride precursors were used with water as the oxidizing reactants and argon acting as a purging gas. The ratio between the alternately laminated HfO_2_ and ZrO_2_ atomic layers (~ 1 nm) was controlled to be 1:1. X-ray reflection and STEM high-angle annular dark-field (HAADF) image indicated the film thickness of 10 nm [29]. Later, TiN and W top electrode layers were deposited by sputtering (PVD-75, Kurt J. Lesker) at room temperature. After photoresist layer patterning of the top electrodes using ultraviolet photolithography (NQX4006, Neutronix-Quintel), the top electrodes were etched into circular shapes with diameters of *l* via ion milling using SF_6_ and O_2_ plasmas with gas flows of 15 and 5 sccm, respectively, at an output power of 50 W for 30 s in a reactive ion etching system (RIE-10NR, Samco, Japan). The amorphous HZO was finally crystallized at 550 °C for 30 s. Near-edge implantation of the oxygen vacancies occurs during etching, as investigated by a stopping and range of ions in matter simulation and X-ray photoelectron spectroscopy (XPS) spectra [24]. The capacitor dimensions were verified using planar-view Scanning Electron Microscope (SEM; Sigma HD, Zeiss) images (Fig. S1a, b).

### Structure Characterization

#### X-ray Diffraction

The phase structure of a large-area TiN/HZO/TiN capacitor without etching was investigated using synchrotron in-plane grazing-incidence X-ray diffraction pattern (XRD) at a wavelength of 0.6887 Å located at the Shanghai Synchrotron Radiation Facility BL17UM [29]. For the study of phase structures within small capacitors, we performed synchrotron X-ray micro-diffraction using an ellipsoidal incident light spot in major and minor axes of 5 and 10 μm, respectively, at a wavelength of 0.6209 Å and an incident energy of 18 keV for illumination time of 30 s.

#### HAADF-STEM Characterization

Cross-sectional TiN/HZO/TiN specimen was prepared for the HAADF-STEM observation using a focused ion beam cutting technique in the FEI Helios G4 system, including low-pressure polishing processes that were performed at 5 and 2 keV. The sample was then treated in a Gatan 691 precision ion polishing system at energies of 1–0.5 keV to remove any residual contamination and damage from the sample surfaces. The STEM experiments were then conducted using a Thermo Fisher Spectra 300 microscope operating at 300 kV with double spherical-aberration (Cs) correctors. HAADF-STEM images were acquired using an annular dark-field image detector that had an inner semi-angle of more than 58 mrad. The probe convergence semi-angle for the microscope was ~ 25 mrad.

### Electrical Characterization

The small-signal capacitance and the loss tangent characteristics of all capacitors were measured using a Precision LCR Meter (Agilent E4980A) operating at an AC amplitude of 0.05 V within the frequency range from 100 Hz to 1 MHz. The dielectric displacement-electric field (*D*-*E*) hysteresis loops at high frequencies were transformed from the measurements of the domain switching current transients versus time [31]. After the application of negative/positive poling voltages to the top electrode of the HZO capacitor, with the bottom electrode remaining grounded, the domain switching/nonswitching currents through an in-series resistor *R* under applied positive switching voltages were observed using an oscilloscope (HDO6054, LeCroy, USA) with 12-bit voltage resolution and a bandwidth of 1 GHz. Through time integration of the current transient, we calculated the charge density and/or the polarization. Square switching pulses were supplied using a two-channel Agilent 81110A pulse generator with adjustable rise/fall times (2 ns-10 ms). The circuit’s resistor–capacitor (*RC*) time constant can be adjusted using an in-series resistance of *R* = 100 Ω − 1 MΩ. For fatigue testing, bipolar square pulses with identical rise/fall times of 2 ns were supplied by the pulse generator with voltage/duration characteristics of ± 1.2 V/50 ns at a repetition frequency of 10 MHz.

## Results and Discussion

### Ultrahigh Dielectric Permittivity

**Fig. 1:**
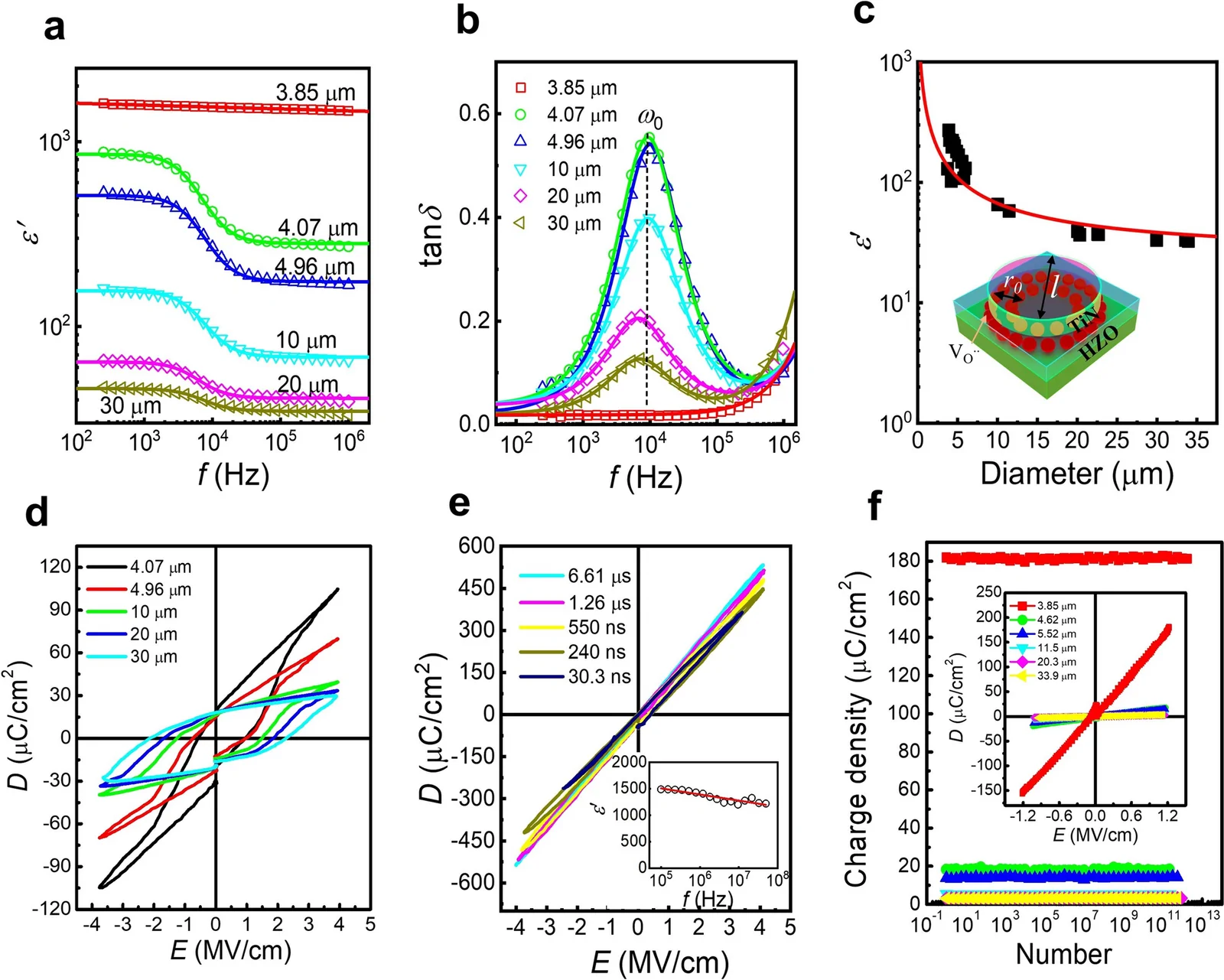
Size-scaling effect on dielectric permittivity. **a, b** Frequency dependences of *ε*′ and tan*δ* for the HZO capacitors of various diameters. The solid lines represent data fittings according to Eqs. (4) − (6). **c** *ε*′-*l* dependence at 1 MHz. The solid line represents the best fit for the data according to Eq. (1) when considering the edging-area contribution under the top electrodes in the geometries illustrated in the inset. **d** *D*-*E* hysteresis loops for the capacitors of various diameters when characterized at 1 MHz. **e** *D*-*E* hysteresis loops at different periodicities for the capacitor in the diameter of 3.85 μm when ferroelectricity disappears. The inset figure shows the high frequency dependence of dielectric permittivity calculated from the slopes of the loops. **f** Cycling number dependences of maximum charge densities at 1.2 V for the size-scaled capacitors when using square fatigue pulses of ± 1.2 V/50 ns at a repetition frequency of 10 MHz. The inset figure shows *D*-*E* hysteresis loops after fatigue

A large amount of oxygen vacancies could appear near the edging region of a TiN/HZO/TiN thin-film capacitor after near-edge ion implantation. These oxygen vacancies (V_O_^⋅⋅^) can diffuse into the inner region in a penetration length of *r*_0_ beneath the top electrode area during final thermal annealing that increases *ε*′ [29]. Figure 1a, b illustrates the frequency (*f*) dependences of *ε*′ and the loss tangent (tan*δ*). *ε*′ increases nonlinearly from 34 to 280 at 1 MHz upon reduction of *l* from 30 to 4.07 μm in Fig. 1a, when *f* < 10 kHz, *ε*′ shows a step-like enhancement accompanying the appearance of dielectric maxima in Fig. 1b. With the continuous shrinkage of *l* down to 3.85 μm, *ε*′ suddenly increases up to 1466 in Fig. 1a along with the disappearance of a dielectric loss peak in Fig. 1b. This implies the ultrahigh *ε*′ transition to occur in a virgin capacitor upon the size-scaling effect without requirement of bipolar high-field cycling [29].

Figure 1c shows the *l* dependence of *ε*′ at 1 MHz. The total capacitance comprises of the capacitances from the edging area of π*r*_0_ (*l* − *r*_0_) and the central area of π (*l/*2 − *r*_0_)^2^ with dielectric permittivities of $\varepsilon^{\prime}_{edge}$ and $\varepsilon^{\prime}_{center}$, respectively, as illustrated in the inset in Fig. 1c. Therefore, we have

1 $$ \varepsilon^{\prime} = \frac{{4r_{0} }}{l}\varepsilon_{edge}^{\prime } + \varepsilon_{center}^{\prime } \left( {l > > r_{0} } \right){.} $$

The solid line in Fig. 1c is the best fit of the data with the assumption of $\varepsilon^{\prime}_{edge}$ = 1466, $\varepsilon^{\prime}_{center}$ = 24 [5–13], and *r*_0_ = 150 nm [29]. Figure 1d shows *D*-*E* hysteresis loops. The loops become more tilted and narrower upon the shrinkage of *l* from 30 to 4.07 μm while the remanent polarization keeps constantly at 17 μC cm^−2^. This is due to the increasing dielectric contribution of edging area ($\varepsilon^{\prime}_{edge}$ >  > $\varepsilon^{\prime}_{center}$) and the resistance of the TiN electrode (Fig. S2a-d). Since* l* >  > *r*_0_, the remanent polarization contribution from the edge area is neglected.

Figure 1e shows the *D*-*E* loops at various periodicities for the capacitor when *l* = 3.85 μm (Fig. S3a-f). All loops are linear, implying the ferroelectric-to-nonferroelectric transition that can occur at the capacitor even if *l* > 2*r*_0_. This finding can relax the restriction on the transitional size of ultrahigh-*ε*′ capacitors from sub-micron to micron. The fundamental physics could be correlated to the high V_O_^⋅⋅^ density within the edging area that triggers ferroelectric-to-nonferroelectric transition within the remaining central area, just like those triggered by bipolar high-field cycling [29]. From the slopes of the loops in Fig. 1e, we calculated dielectric permittivities at different frequencies, as shown in the inset (Fig. S3f), in agreement with those in Fig. 1a. After the ferroelectric-to-nonferroelectric transition, the stored charge density increases by more than 10 times (Fig. S4a − d). The storing charge density of the nonferroelectric capacitor at an operating voltage/time of 1.2 V/50 ns is 185 μC cm^−2^ at cycle numbers of more than 10^12^ without inducing dielectric breakdown, as shown in the in Fig. 1f, much higher than 3.2 − 18 μC cm^−**2**^ of other ferroelectric capacitors of various diameters (Fig. S5a-c). The huge charge density is extremely attractive for high-density DRAM and CMOS applications [2, 3].

**Fig. 2:**
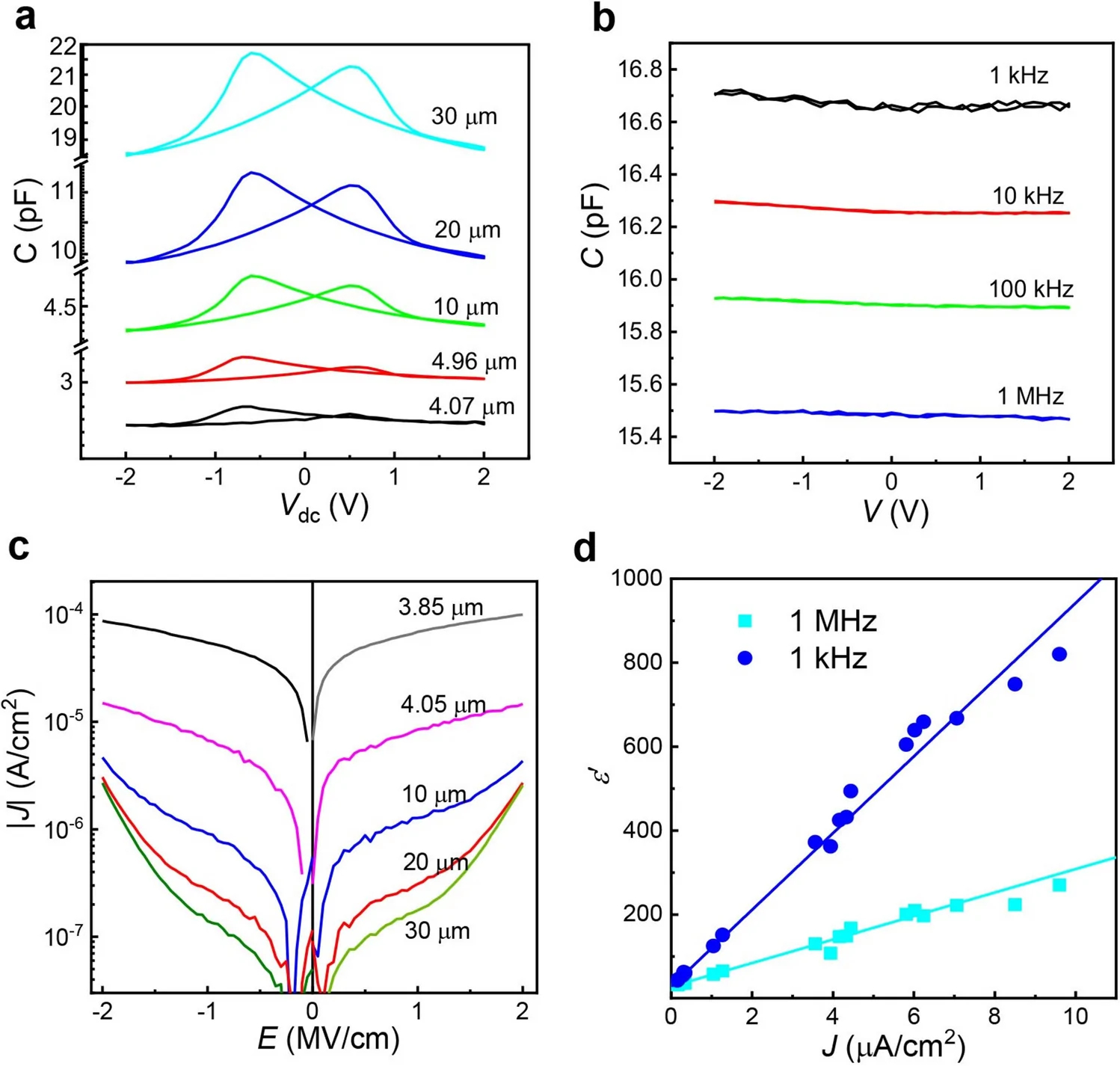
*C* − *V* loops in ferroelectric and nonferroelectric capacitors. **a** Typical *C*-*V* loops at 1 MHz for ferroelectric capacitors of various diameters. **b** *C*-*V* loops at different frequencies for a nonferroelectric capacitor in the diameter of 3.85 μm. **c** log|*J*|− *E* curves for the capacitors of various diameters. **d** Leakage current density (1 MV cm^−1^) dependences of dielectric permittivities at different frequencies fitted by two solid lines

For the discrimination of ferroelectric and nonferroelectric properties of the capacitors, we measured capacitance–voltage (*C*-*V*) curves. All loops when *l* = 4.07 − 30 μm demonstrate a typical “butterfly” shape of a ferroelectric capacitor, as shown in Fig. 2a. But the loop is linear irrespective of the applied voltages at different frequencies when *l* = 3.85 μm, as shown in Fig. 2b (Fig. S6a, b). This confirms the ferroelectric-to-nonferroelectric transition upon the size-scaling effect.

To build up relationship of the enhanced dielectric permittivity in Fig. 1a with increasing fraction of the near-edge area rich with oxygen vacancies, we measured electric field dependences of leakage current density (*J*) of the size-scaled capacitors, as shown in Fig. 2c. The leakage current density increases quickly with the shrinkage of *l* from 30 to 3.85 μm due to the increasing V_O_^⋅⋅^ contribution within the edging area. Subsequent *ε*′-*J* plots either at low or high frequencies (1 kHz or 1 MHz) are linear, as shown by the solid-line fits of the data in Fig. 2d, and the two plots both intercept with the *ε*′-axis in an intrinsic dielectric permittivity of 24 without the V_O_^⋅⋅^ contribution [5−13]. Therefore, the linear plots build up a close relationship among *ε*′, *J* and V_O_^⋅⋅^. The nonlinear *J* − *E* curves can be fitted by a power law in the form of *J* ∝ *E*^n^, where *n* is a coefficient. *n* varies from 0.48 to 0.84 upon the *l* shrinkage from 30 to 3.85 μm across the edge area but keeps constant at 8.1 across the central area (Fig. S7a, b).

### Dielectric Dispersion

The dielectric relaxation of oxygen vacancies within the near-edge area can be described using the classical Debye equation given by [32]:

2 $$ \varepsilon \left( \omega \right) = \varepsilon_{\infty } + \frac{{\varepsilon_{0} - \varepsilon_{\infty } }}{1 + i\varpi \tau }, $$

where *τ* is relaxation time of oxygen vacancies, and *ε*_∞_ and *ε*_0_ are dielectric permittivities at the highest and lowest frequencies (*ω* = 2π*f*), respectively. Therefore, the dielectric loss peak in Fig. 1b can appear at the frequency:

3 $$ \omega_{0} = \sqrt {\frac{{\varepsilon_{0} }}{{\varepsilon_{\infty } }}} \frac{1}{\tau }. $$

With the data in Fig. 1a, b, we calculated *τ* between 25 and 29 μs. In consideration of the partial oxidation of the TiN top electrode during thermal crystallization of the HZO thin film [32], Eq. (2) is modified to read:

4 $$ \varepsilon \left( \omega \right) = \frac{{\varepsilon_{0} + i\omega \tau \varepsilon_{\infty } }}{{\left( {1 - \omega^{2} \tau R_{i} C_{0} \varepsilon_{\infty } } \right) + i\omega \tau }}(\tau > > R_{i} C_{0} ), $$

where *C*_0_ = *ε*_v_*S*/*d*, *d* is the film thickness, *ε*_v_ is the vacuum permittivity, and *R*_i_ is the resistance of TiN electrodes. The solid lines in Fig. 1a, b are the best fits of the data based on the consideration that *ε* = *ε*′ − i*ε*″, tanδ = *ε*″/*ε*′ and *R*_i_ = 0.76 − 0.98 kΩ [29]. After ferroelectric-to-nonferroelectric transition, a universal law can then be used to describe the dielectric dispersion in the following form of [33]:

5 $$ \varepsilon \left( \omega \right)\, \propto  \, \left( {i\omega } \right)^{n - 1} $$

and

6 $$ \tan \delta = \omega \tau^{\prime} + {\mathrm{ctan}}\frac{n\pi }{2} $$

where 0 < *n* ≤ 1 and *τ*′ = *CR*_i_. The solid lines shown in Fig. 1a, b represent the best fits for the data when *l* = 3.85 μm according to Eqs. (5) and (6) from which we derived *n* = 0.90.

### Phase Structure

**Fig. 3:**
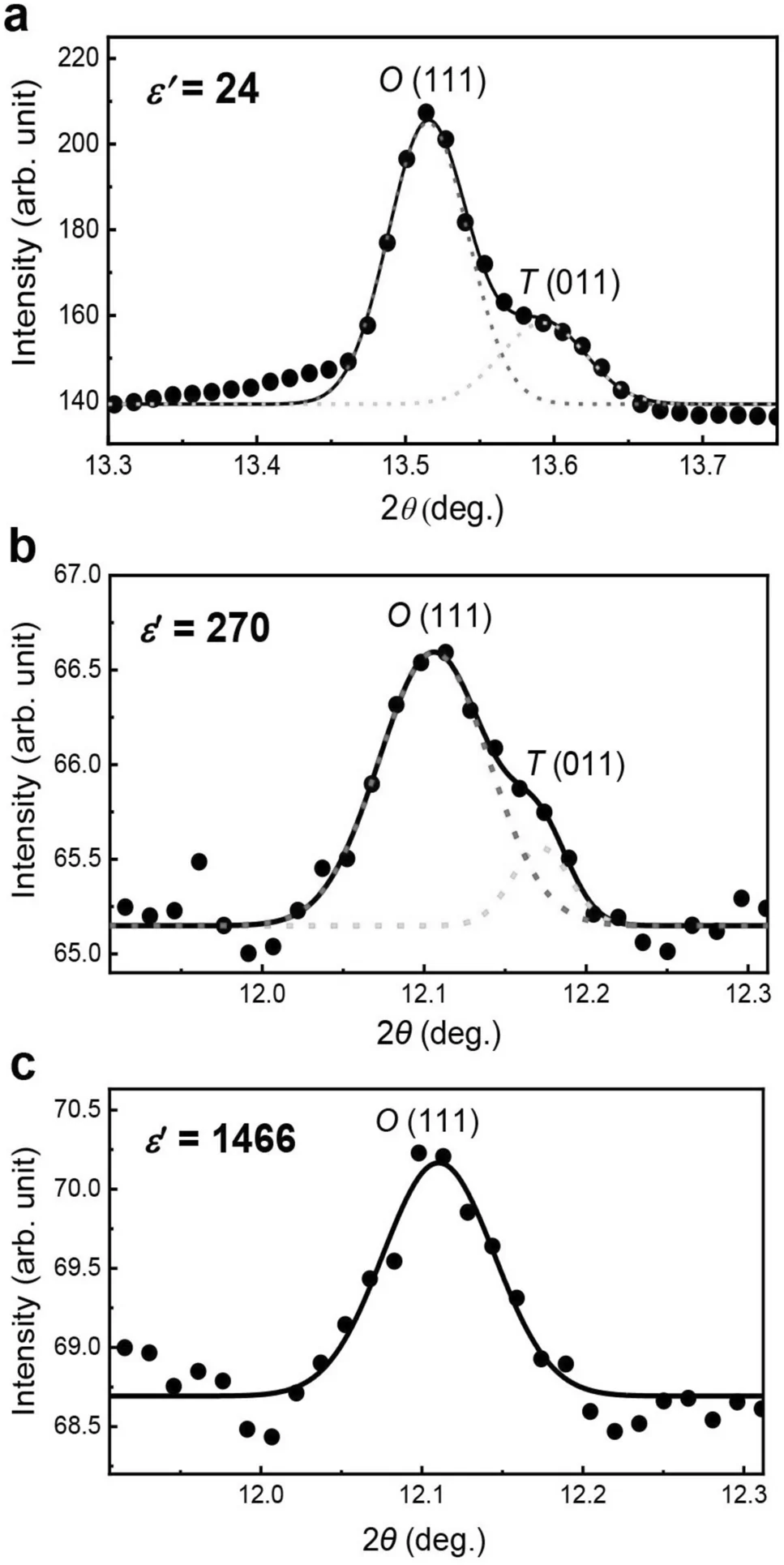
XRD patterns. **a** Synchrotron in-plane grazing-incidence diffraction pattern for a large-area TiN/HZO/TiN capacitor using a synchrotron radiation source at a wavelength of 0.6887 Å. **b, c** Synchrotron X-ray micro-diffraction patterns of small capacitors in diameters of 4.07 μm and 3.85 μm with/without ferroelectricity, respectively, using a synchrotron radiation source at a wavelength of 0.6209 Å. From solid and dashed line fits of the peaks using the Gaussian function, we calculated area ratios of 0.76:0.24, 0.87:0.13, and 1:0 for O (111) and T (011) reflections with increasing dielectric permittivities from 24 to 1466, respectively. All patterns were corrected using W (110) reflections of top electrodes

Synchrotron in-plane grazing-incidence XRD for a large-area TiN/HZO/TiN capacitor revealed a mixture of O, T and M phases [29]. Figure 3a shows the overlapping O (111) and T (011) reflections with the area ratio of O:T = 0.77:0.23 after Gauss functional fittings of all peaks by smooth dashed lines. For micron-sized capacitors, we measured their synchrotron X-ray micro-diffraction patterns in Fig. 3b, c. With the shrinkage of *l* down to 4.07 μm, the T (011) reflection weakens in Fig. 3b with O: T = 0.87:0.13, and completely disappears from Fig. 3c (*l* = 3.85 μm) when the ultrahigh *ε*′ transition occurs. It seems that both O (111) and T (011) lattices expand by 0.68% in the etched HZO capacitors after near-edge ion injection (Fig. S8a-c). In contract, the M (011) reflections from all capacitors are nearly the same (Fig. S9a, b).

**Fig. 4:**
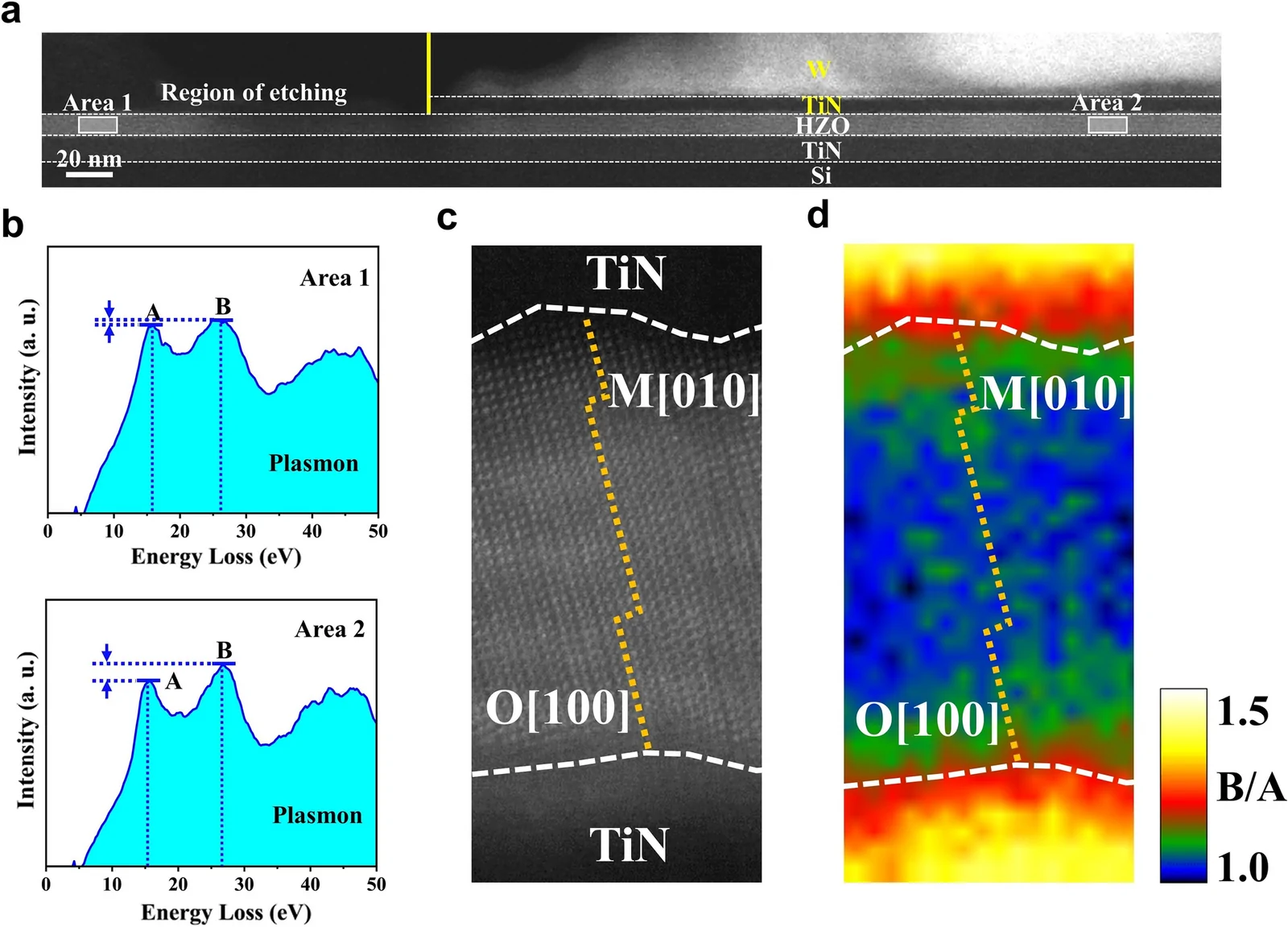
Phase structure and oxygen vacancy distribution. **a** Low-magnification HAADF-STEM cross-sectional image of a 300 nm-sized TiN/HZO capacitor. **b** Typical O K EELS spectra within areas 1 and 2 in **a**. **c** HAADF-STEM image near the phase boundary below the TiN top electrode. **d** EELS mapping of the B/A distribution in **c**

Generally the T phase can appear as the interfacial layer between the top TiN electrode and the O-phase grain [34]. The low-*ε*′ T phases affiliated with ultrahigh-*ε*′ O grains in the ultrathin films could cause the overall permittivity to be one to two orders of magnitude lower than expected [37−44]. However, the T → O transition occurs firstly during polarization fatigue [43], and then the periodical V_O_^⋅⋅^ accumulation near top and bottom electrodes during fatigue can promote ultrahigh *ε*′ transition within the entire O − phase grain (Fig. S10a, b) [29]. To understand the effect of the possible V_O_^⋅⋅^ accumulation on ultrahigh-*ε*′ transition without fatigue here, we performed STEM − HAADF imaging of phase structures from the edging to the central regions across a 0.3-μm-sized capacitor (*l* = 2*r*_0_) in Fig. 4a and traced the distribution of the oxygen vacancies by performing an electron energy loss spectroscopy (EELS)-based imaging analysis of the O K edge in Fig. 4b. The O K energy-loss near-edge structure of the HZO presents double peaks, labelled A and B, with an energy difference of ~ 11 eV, which are attributed to the O 2*p* orbitals hybridized with the Hf (or Zr) $d_{{e_{g} }}$ and $d_{{t_{2g} }}$ orbitals, respectively [44]. The relative sharpnesses and intensities of these two peaks vary with the content of oxygen vacancies (B/A) [24]. It seems that oxygen vacancies increase from the edging to central area because of the increasing B/A ratio in Fig. 4b. For understanding of the oxygen vacancy distribution within the central area, Fig. 4c, d shows the HAADF-STEM image and EELS mapping of the B/A ratio over two adjacent O [100] and M [010] grains within the Area 2. There are notable increases in the B/A ratio from 1.0 to 1.1 at interfaces near top and bottom electrodes as well as the phase boundary (green areas), implying oxygen vacancy accumulation within these regions. Meantime, there is no observable interfacial *T* phase affiliated with the O grain in Fig. 4c (Fig. S10b). It seems that the interfacial oxygen vacancy accumulation within the pure O-phase grain promotes the ultrahigh-*ε*′ transition.

For a smaller capacitor, dielectric contribution from the edging region rich with oxygen vacancies becomes more important. The first-principles calculations of the O moving energy barrier through the minimum energy path in a 96-atom supercell is as low as 0.51 eV because of partial nonstoichiometry in the lattice and dangling bonds at cation atoms. With the input of two additional oxygen vacancies in the supercell, the O moving barrier disappears in the lowest transformation-state energy. This vanished energy barrier energetically favors spontaneous and directional O movement during V_O_^⋅⋅^ ordering. Meantime, the calculated state-transformation energy barrier between two opposite polarization states is also sensitive to the number of oxygen vacancies. With the introduction of 1–8 three-coordinated oxygen vacancies into the 96-atom supercell, the energy barrier decreases progressively from 2.57 to 1.03 eV. During V_O_^⋅⋅^ ordering, more oxygen vacancies are involved, thus lowering the energy barrier further until the formation of a new defective orthorhombic phase where multiple polar regions enhance ε′ significantly [29].

## Conclusions

We observed the *ε*′ enhancement upon the lateral shrinkage of the HZO capacitor. The high *ε*′ arises from the near-edge ion implanted region of the capacitor rich with oxygen vacancies. When the capacitor diameter is shrunk down to 3.85 μm, the ferroelectric-to-nonferroelectric transition occurs with the appearance of an ultrahigh *ε*′ of 1466 independent of the applied voltage. The subsequent studies of synchrotron X-ray micro-diffraction patterns and STEM and EELS images show the preferred oxygen vacancy accumulation within the interfacial layers as well as the phase boundary. The large amount of accumulated oxygen vacancies can reduce the energy barriers for spontaneous and directional O movements during ferroelectric-to-nonferroelectric transition [29]. This finding advances the fabrication technique of an ultrahigh-*ε*′ material without bipolar high-electric-field cycling. A parallel connection of many ultrahigh-*ε*′ capacitors could extend the total capacitor area. More attentions should be paid to the reduction of the leakage current density of the HZO capacitor in the future.

## Supplementary Information

Below is the link to the electronic supplementary material.

Supplementary file1 (DOCX 4009 KB)
