# Supplementary material for: Ultrahigh Dielectric Permittivity of a Micron-Sized Hf0.5Zr0.5O2 Thin-Film Capacitor After Missing of a Mixed Tetragonal Phase
Source: Nanomicro Lett. 2025 Jul 18;18:6. doi: 10.1007/s40820-025-01841-x (PMC12274175; doi:10.1007/s40820-025-01841-x)
Supplement: Supplementary file 1 — Supplementary file1 (DOCX 4009 KB) [file 40820_2025_1841_MOESM1_ESM.docx]

Supporting Information for

**Ultrahigh Dielectric Permittivity of a Micron-Sized Hf_0.5_Zr_0.5_O_2_ Thin-Film Capacitor after Missing of a Mixed Tetragonal Phase**

Wen Di Zhang^1^, Bing Li^2^, Wei Wei Wang^3^, Xing Ya Wang^3^, Yan Cheng^4,^* and An Quan Jiang^1,^*

^1^ College of Integrated Circuits and Micro/Nano Electronics Innovation, Fudan University, Shanghai 200433, P. R. China

^2^ Center for Transformative Science, ShanghaiTech University, Shanghai 201210, P. R. China

^3^ Shanghai Advanced Research Institute, Chinese Academy of Sciences, Shanghai 201204, P. R. China

^4^ Key Laboratory of Polar Materials and Devices (MOE), Department of Electronics, East China Normal University, Shanghai 200241, P. R. China

*Corresponding authors. E-mail: [aqjiang@fudan.edu.cn](mailto:aqjiang@fudan.edu.cn) (An Quan Jiang); [ycheng@ee.ecnu.edu.cn](mailto:ycheng@ee.ecnu.edu.cn) (Yan Cheng)

**Supplementary Figures**


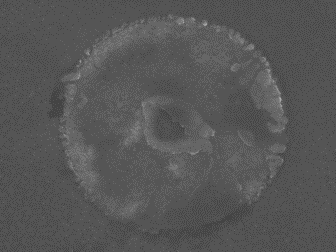


**3.85 μm**

1 μm


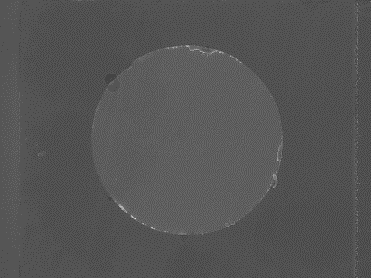


**30 μm**

10 μm

**b**

**a**

**Fig. S1** Capacitor size. **a** **b** Planar SEM photographs of top electrodes for state-of-the-art HZO capacitors


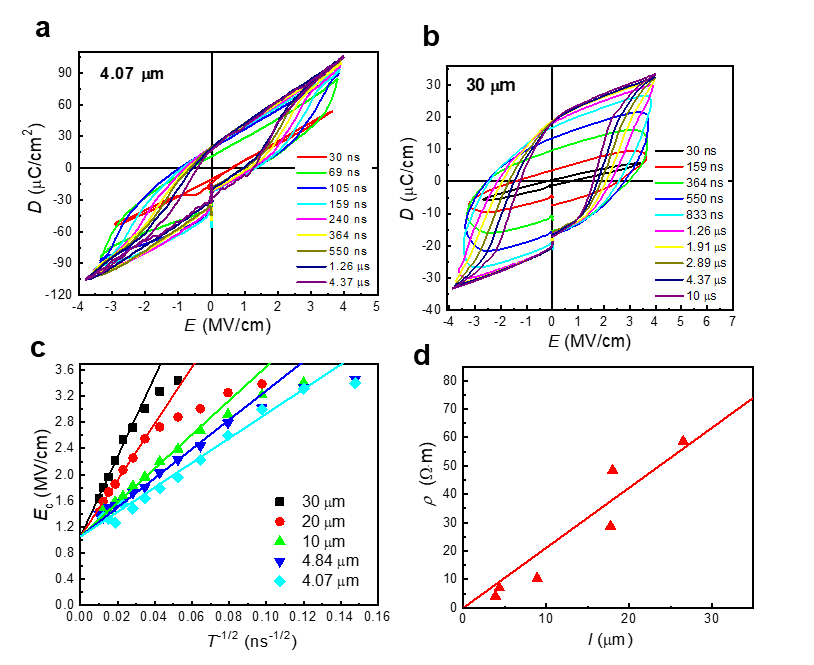


**Fig. S2** *D*-*E* hysteresis loops of size-scaled capacitors. **a** **b** *D*-*E* hysteresis loops at different periodicities for two ferroelectric capacitors in different diameters. **c** *E*_c_-*T*^-1/2^ dependences of the capacitors of various diameters. The solid lines are the linear fits of the data according to Eq. S1. **d** The *ρ*-*l* dependence fitted by the solid line according to Eq. S2.


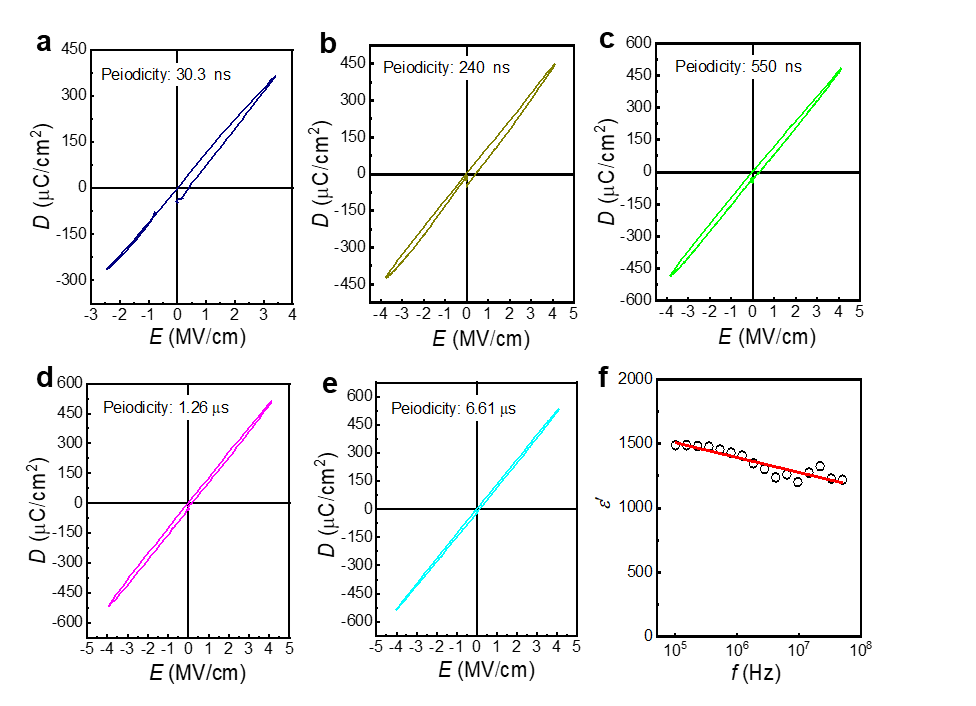


**Fig. S3** Ultrahigh dielectric permittivity. **a-e** *D*-*E* hysteresis loops at different periodicities for the capacitor in the diameter of 3.85 μm when ferroelectricity disappears. **f** High frequency dependence of dielectric permittivity calculated from the slopes of the loops


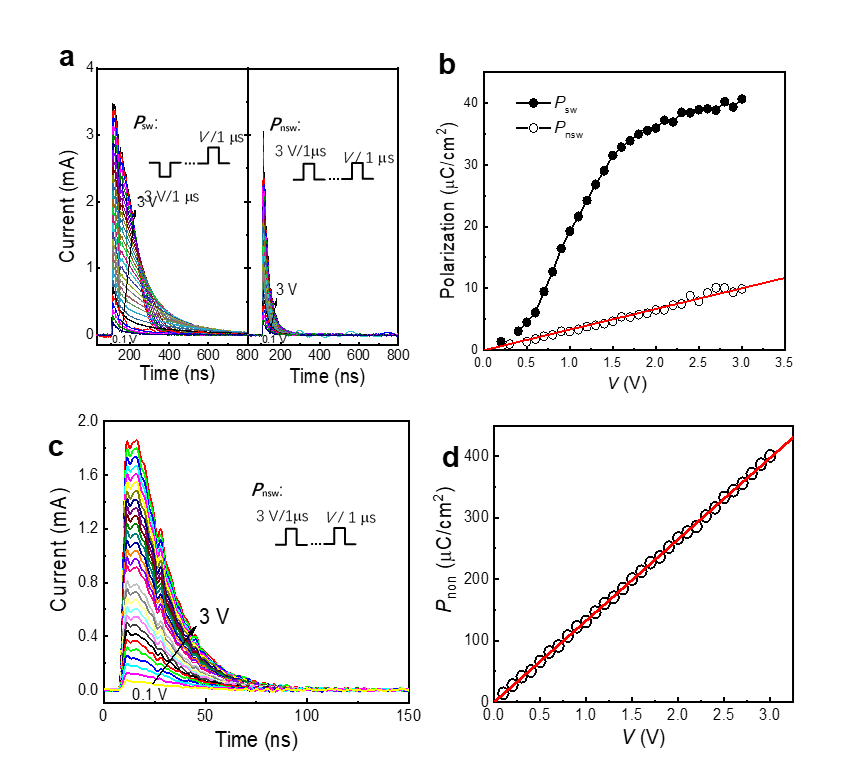

**Fig. S4** Domain switching current transients. **a** Switching/nonswitching current transients versus time with increasing *V* from 0.1 to 3 V in steps of 0.1 V for a ferroelectric capacitor in the diameter of 30 μm using the pulse sequences sketched in the insets. **b** Voltage dependences of switching/nonswitching polarizations (*P*_sw_ and *P*_non_) in **a**. **c** Nonswitching current transients versus time for a nonferroelectric capacitor in the diameter of 3.85 μm with increasing *V* from 0.1 to 3 V in steps of 0.1 V using the pulses sequence sketched in the inset. **d** Voltage dependence of *P*_non_ in **c** fitted by the solid line

**a**

**b**

**c**

**Fig. S5** Size-scaling effect on storing charge density. **a** Cycling number dependences of maximum charge densities at 1.2 V for the sized-scaled capacitors after poling using the pulses sketched in the inset when using square fatigue pulses of ±1.2 V/50 ns at a repetition frequency of 10 MHz. **b** *D*-*E* hysteresis loops for the capacitors of various diameters after fatigue when characterized at 10 MHz. **c** Cycling number dependences of remanent polarization (*P*_r_) for the sized-scaled capacitors when using square fatigue pulses of ±4 V/500 ns at a repetition frequency of 1 MHz. The inset figure shows *D*-*E* hysteresis loops after different fatigue cycles for a small capacitor in the diameter of 5.23 μm until the occurrence of dielectric breakdown, where the parenthetic value shows the zoom-in dielectric displacement.

-1.2 V/50 ns

1.2 V /50 ns

**TiN**

**Fig. S6** Dielectric loss for the ultrahigh-*ε*′ capacitor. **a** DC voltage dependences of the dielectric loss at different frequencies for a nonferroelectric capacitor in the diameter of 3.85 μm. **b** Oscillating voltage dependences of the capacitance at different frequencies.

**b**

**a**

**Fig. S7** Leakage current densities of size-scaled capacitors. **a** Electric field dependences of leakage current density for the capacitors of various diameters fitted by the solid lines according to Eq. S3. **b** The *n*-*J*_edge_ dependence for the edging area fitted by the solid line

**a**

**b**

**Fig. S8** XRD patterns. **a** Synchrotron in-plane grazing-incidence diffraction pattern for a large-area TiN/HZO/TiN capacitor using a synchrotron radiation source at a wavelength of 0.6887 Å. **b c** Synchrotron X-ray micro-diffraction patterns of small capacitors in diameters of 4.07 μm and 3.85 μm with/without ferroelectricity, respectively, using a synchrotron radiation source at a wavelength of 0.6209 Å. From solid and dashed line fits of the peaks using the Gaussian function, we calculated area ratios of 0.76:0.24, 0.87:0.13 and 1:0 for O (111) and T (011) reflections with increasing dielectric permittivities from 24 to 1466, respectively.

**b**

**a**

**c**

**b**

**a**

**Supplementary Results and Discussion**

**Fig. S10** Phase structure. **a** The typical O grain affiliated with a thin layer of the T phase near the top electrode within a large-area TiN/HZO/TiN capacitor when ε′ =24. The data were adapted from Ref. 5. **b** The HAADF-STEM image of a typical O-phase grain within the ultrahigh-*ε*′ TiN/HZO/TiN capacitor without ferroelectricity. The inset shows the atomic modeling of the O [010] orientation

**Fig. S9** XRD patterns. **a** **b** Synchrotron X-ray micro-diffraction patterns of two capacitors in diameters of 4.07 μm and 3.85 μm with/without ferroelectricity, respectively, using a synchrotron radiation source at a wavelength of 0.6209 Å

**b**

**a**


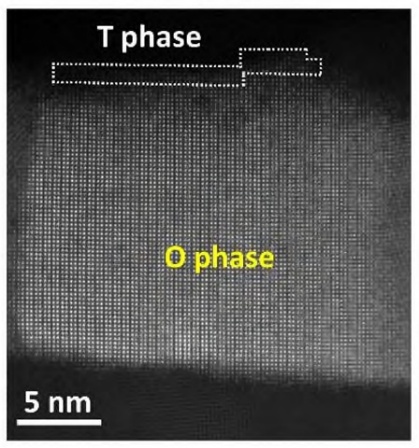

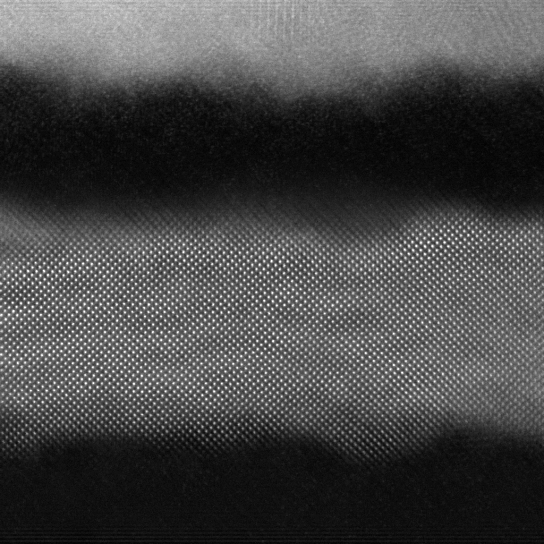


**O phase**

5 nm


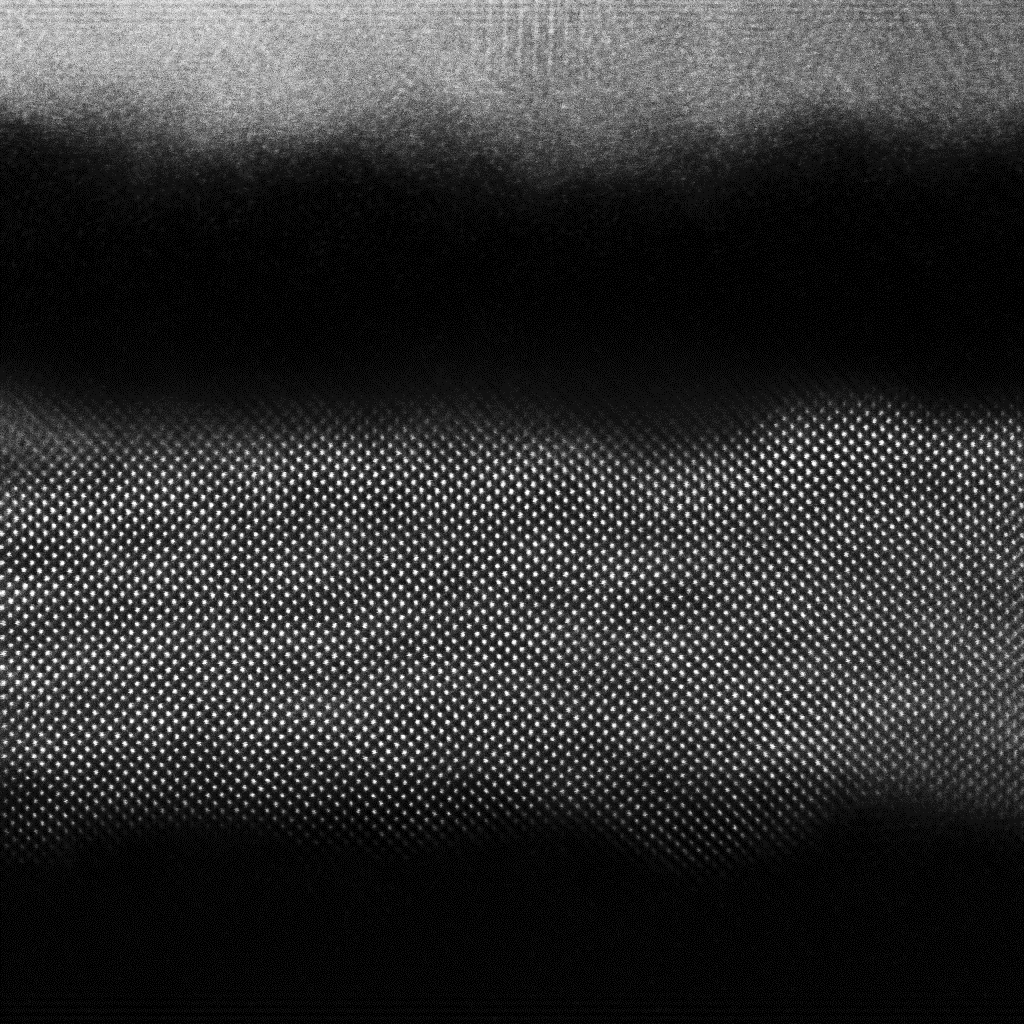


**x**


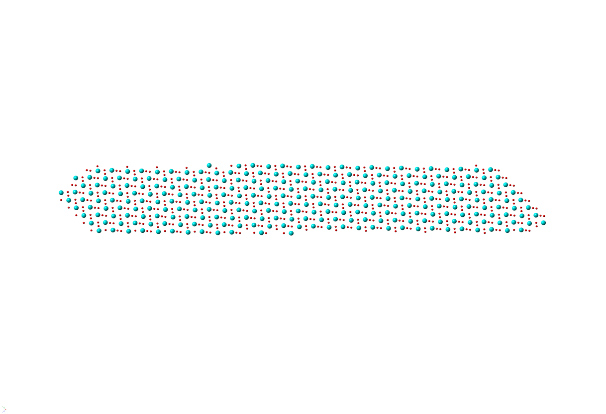

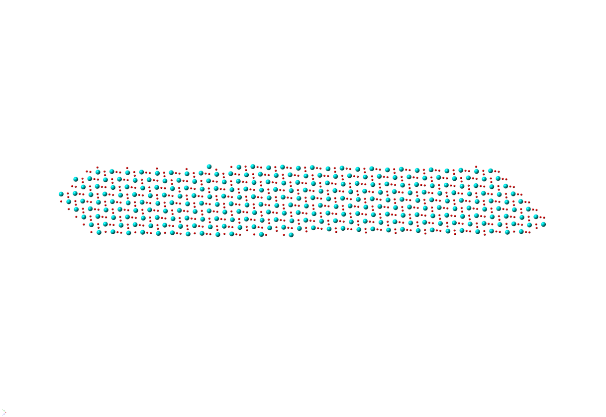


O [010]

1 nm

**Note S1 Effect of the TiN resistance on the domain switching speed**

Continuous Hf_0.5_Zr_0.5_O_2_ thin films were grown by ALD at 200 °C on a Si substrate coated with 10-nm-thick TiN bottom electrodes. After etching of the top electrodes. The thermal annealing process was performed at 550°C for 30 s in a N_2_ atmosphere to enable crystallization of the HZO. Near-edge implantation of the oxygen vacancies occurs during etching. The capacitor sizes were determined by reactive ion etching of top electrodes (W/TiN) after ultraviolet photolithography patterning of the Cr shallow masks and were calibrated carefully from SEM photographs, as shown in Fig. S1a, b. Figure S2a, b shows *D*-*E* hysteresis loops at different periodicities for two ferroelectric capacitors of various diameters (*l*). the coercive field (*E*_c_) enlarges with either the reduction of *T* or the increase in *l*. This is due to the partial oxidation of TiN top electrode in contact with HZO during thermal crystallization that increases the electrode resistivity (*ρ*_on_) [S1]. *E*_c_ determined in the *D*-*E* hysteresis loop can be described in the form of [S2]

$E_{c}=E_{c0}+\sqrt{8P_{r}E_{a}\rho_{on}/T}$ (S1)

where *E*_c0_ is the intrinsic coercive field independent of *T* and *l*, *E*_a_ is the amplitude of the applied electric field, and *P*_r_ is the remanent polarization. Figure S2c shows *E*_c_−*T*^-1/2^ dependences for the capacitors of various *l*. All plots are almost linear and converge into *E*_c0_ = 1.06 MV/cm as *T*^-1/2^→0, as shown by the solid line fits of the data. From the slopes of linear fits, we estimated *ρ*_on_ against *l* with the assumption of *E*_a_ = 4 MV/cm, and *P*_r_ = 17 μC/cm^2^, as plotted in Fig. S2d. *ρ*_on_ reduces almost linearly against *l* in the form of [S2]:

$\rho_{\text{on}}=R_{on}l$ (S2)

where *R*_on_ is the resistance of the interfacial layer. From the solid-line fit of the data, we estimated *R*_on_ = 2.11 MΩ. Figure S3c shows the separate plots of *D*-*E* hysteresis loops and dielectric dispersion at different periodicities for the capacitor in the diameter of 3.85 μm in main text of Fig. 1e more clearly.

**Note S2 Domain switching current transients versus time**

From positive-up-negative-down (PUND) testing, it is possible to measure domain switching and nonswitching current transients versus time under different applied voltages for a ferroelectric capacitor in Fig. S4a, c using the pulse sequences sketched in the insets. After time integration of the current transient, we got voltage dependences of switching and nonswitching polarizations (*P*_sw_ and *P*_non_), as plotted in Fig. S4b, d, and 2*P*_r_ = *P*_sw_-*P*_non_. For a nonferroelectric capacitor when *l* = 3.85 μm, the two current transients of *P*_sw_ and *P*_non_ are nearly the same, as shown in Fig. S4c. Meantime, its *P*_non_−*V* dependence is linear, as shown in Fig. S4d. From the slope of a linear fit, we derived that *ε*′ = 1490, roughly in agreement with the measurement of an impedance analyser in Fig. 1a. Figure S5a shows the fatigue number dependences of maximum charge densities for the size-scaled capacitors using square fatigue pulses of ±1.2 V/50 ns at a repetition frequency of 10 MHz. Though the storing charge density increases from 3.2 to 18 μC/cm^2^ upon downscaling of the ferroelectric capacitor size from 33.5 to 4.5 μm, they are much lower than 185 μC/cm^2^ of a nonferroelectric capacitor in the diameter of 3.85 μm in Fig. 1f. The charge densities of all ferroelectric capacitors keep constant against the fatigue number, and all domains become unswitched at +/-1.2 V, as implied from the linear *D*-*E* hysteresis loops in Fig. S5b. Once the fatigue voltage increases up to 4 V, the dielectric breakdown occurs when *N* = 10^7^−10^8^ among the ferroelectric capacitors in the diameters of 5.23-20.1 μm in Fig. S5c before the occurrence of ultrahigh *ε*′ transition. This highlights the advantage of ultrahigh *ε*′ transition within a virgin 3.85 μm-sized capacitor without requirement of high-field cycling.

**Note S3 Voltage-independent ultrahigh *ε*′**

In most ferroelectrics, both *ε*′ and tan*δ* have nonlinear dependence of either the amplitude of the applied AC voltage (*V*_­osc_) or the DC voltage (*V*) due to domain-wall pinning and depinning by homogeneously and inhomogeneously distributed oxygen vacancies (Fig. 2a) [S3, S4]. Figure S6a, b shows tan*δ*−*V* and *C*−*V*_osc_ dependences at different frequencies for a nonferroelectric capacitor when *l* = 3.85 μm, respectively, where *C* depends less on either *V* or *V*_osc_. These observations confirm the ferroelectric-to-nonferroelectric transition.

**Note S4 Leakage currents in HZO capacitors**

Figure S7a shows the electric field dependences of leakage current density for the capacitors of various diameters. The total leakage current density comprises leakage currents from near-edge and central areas that both obey the power laws in the form of

$J\left(E\right)=J_{edge}E^{n}+J_{center}E^{m}$ (S3)

where *J*_edge_ and *J*_center_ are constants and *n* and *m* are the coefficients. After solid line fits of the data in Fig. S7a, we found that *n* from the edging area ranges from 0.48 to 0.84 with the reduction of *l* from 30 to 3.85 μm and can be fitted by the relationship of *n* ∝ *J*_edge_^-0.092^, as shown by the solid-line fit of the data in Fig. S7b. In comparison, the leakage current density from the central area is nearly constant with *m* = 8.1 for all capacitors in the diameters of 4.05−30 μm when *r*_0_ << *l*.

**Note S5 Phase structure**

Figure S8 (a)-(c) are interplanar spacing scanning XRD patterns transformed from Fig. 3a-c in main text. From these patterns, it understood that both O (111) and T (011) lattices expand by 0.68 % in the etched HZO capacitors. This lattice expansion could be correlated to the reduced capping effect of top electrodes in small capacitors and near-edge ion injection that creates more oxygen vacancies. Figure S9a, b shows synchrotron X-ray micro-diffraction patterns of two capacitors in diameters of 4.07 μm and 3.85 μm, respectively. It seems that the phase structure of the ferroelectric capactor is the mixture of O, T and M phases. But the T phase disappears from the latter nonferroelectric capacitor (Fig. 8c) when the ultrahigh *ε*′ transition occurs.

In a ferroelectric HZO capacitor, a thin T-phase layer affliated to the O grain was always found at the top interface with the thickness ranging from 0.75 nm to 1 nm, as shown in Fig. S10a [S5]. The low-*ε*′ T phase in series with the ultrahigh-*ε*′ O phase can reduce the overall permittivity by one to two orders of magnitude. In contrast, the T layer disappears from the nonferroelectric capacitor after the occurrence of ultrahigh *ε*′ transition. Figure S10b show the typical STEM-HAADF image of the [010] O grain within the central area in Fig. 4a (Area 2), where there is no interfacial T phase, in agreement with the XRD observation in Fig. 3c.

**Supplementary References**

1. W. Hamouda, A. Pancotti, C. Lubin, L. Tortech, C. Richter et al., Physical chemistry of the TiN/Hf_0.5_Zr_0.5_O_2_ interface. J. Appl. Phys. **127**(6), 064105 (2020). <https://doi.org/10.1063/1.5128502>
2. W.D. Zhang, A.Q. Jiang, Size-scaling effect on domain switching time and coercive field of TiN/Hf_0.5_Zr_0.5_O_2_/TiN thin-film capacitors. IEEE Trans. Electron Devices **70**(12), 6324–6328 (2023). <https://doi.org/10.1109/TED.2023.3325417>
3. L.M. Garten, S. Trolier-McKinstry, The field induced E31, f piezoelectric and Rayleigh response in barium strontium titanate thin films. Appl. Phys. Lett. **105**(13), 132905 (2014). <https://doi.org/10.1063/1.4897299>
4. C. Filipič, A. Levstik, Z. Kutnjak, Analytical behavior of dielectric nonlinearity in PbMg_1/3_Nb_2/3_O_3_relaxor ferroelectric. Ferroelectrics **257**(1), 63–68 (2001). <https://doi.org/10.1080/00150190108016282>
5. Y. Cheng, Z. Gao, K.H. Ye, H.W. Park, Y. Zheng et al., Reversible transition between the polar and antipolar phases and its implications for wake-up and fatigue in HfO_2_-based ferroelectric thin film. Nat. Commun. **13**(1), 645 (2022). <https://doi.org/10.1038/s41467-022-28236-5>
